# Unappreciated Role of LDHA and LDHB to Control Apoptosis and Autophagy in Tumor Cells

**DOI:** 10.3390/ijms20092085

**Published:** 2019-04-27

**Authors:** Kaja Urbańska, Arkadiusz Orzechowski

**Affiliations:** 1 Department of Morphological Sciences, Warsaw University of Life Sciences—SGGW, Nowoursynowska 159, 02-776 Warsaw, Poland; kaja_urbanska@sggw.pl; 2 Department of Physiological Sciences, Warsaw University of Life Sciences—SGGW, Nowoursynowska 159, 02-776 Warsaw, Poland

**Keywords:** lactate dehydrogenase A (LDHA), lactate dehydrogenase B (LDHB), apoptosis, autophagy, tumor

## Abstract

Tumor cells possess a high metabolic plasticity, which drives them to switch on the anaerobic glycolysis and lactate production when challenged by hypoxia. Among the enzymes mediating this plasticity through bidirectional conversion of pyruvate and lactate, the lactate dehydrogenase A (LDHA) and lactate dehydrogenase B (LDHB), are indicated. LDHA has a higher affinity for pyruvate, preferentially converting pyruvate to lactate, and NADH to NAD^+^ in anaerobic conditions, whereas LDHB possess a higher affinity for lactate, preferentially converting lactate to pyruvate, and NAD^+^ to NADH, when oxygen is abundant. Apart from the undisputed role of LDHA and LDHB in tumor cell metabolism and adaptation to unfavorable environmental or cellular conditions, these enzymes participate in the regulation of cell death. This review presents the latest progress made in this area on the roles of LDHA and LDHB in apoptosis and autophagy of tumor cells. Several examples of how LDHA and LDHB impact on these processes, as well as possible molecular mechanisms, will be discussed in this article. The information included in this review points to the legitimacy of modulating LDHA and/or LDHB to target tumor cells in the context of human and veterinary medicine.

## 1. Lactate Dehydrogenases and Lactate

Lactate dehydrogenase [(S)-lactate:NAD^+^ oxidoreductase; LDH;1; EC 1.1.1.27] comes from a family of NAD^+^-dependent enzymes. Active LDH is a homo- or heterotetramer molecule, which is assembled by an association of two different subunits: M and H, named as such, since they were originally detected in skeletal muscle (M) and heart (H). In human beings, these polypeptide subunits are encoded by two structurally distinct genes: *LDHA* (M) and *LDHB* (H) [1,2]. The *LDHA* gene is located on chromosome 11, while *LDHB* is found on chromosome 12. However, chromosomes 1, 2, 4, 9, and 10 apparently contain *LDHA* gene-related sequences, whereas *LDHB* gene-related sequences are found in the X chromosome and chromosome 13 [3].

**Figure 1:**
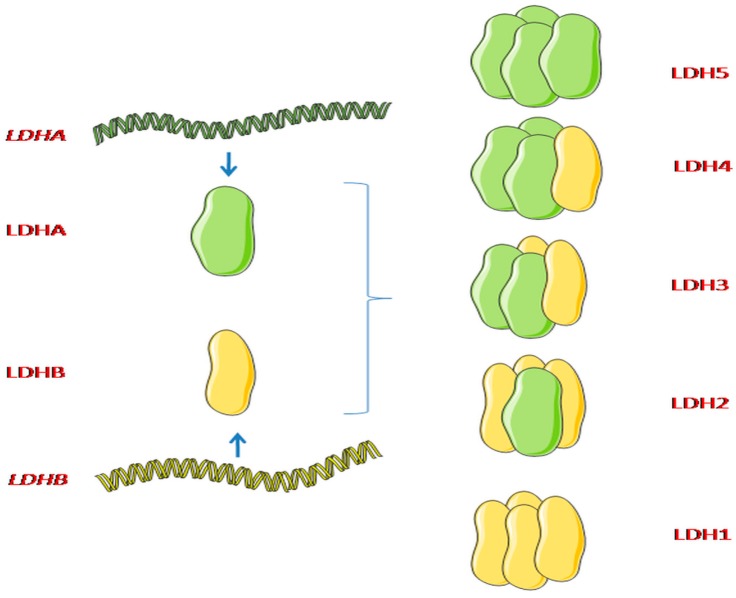
Lactate dehydrogenase (LDH) subunits and their combinations. Lactate dehydrogenase (LDH) consists of two different subunits: Lactate dehydrogenase A (LDHA) and lactate dehydrogenase B (LDHB). LDHA and LDHB can be assembled into combinations: LDH1 is composed from four LDHB subunits; LDH2 contains three LDHB subunits and one LDHA; LDH3 has two LDHB/LDHA subunits; LDH4 possesses one LDHB subunit and three LDHA subunits; while LDH5 is composed from four LDHA subunits [4]. Figure conception adapted from Doherty et al., (2013). Graphical elements adapted from Servier Medical Art.

The association of the subunits M and H is random. It generates five isoenzymes LDH1 to LDH5, differing in their subunit proportions and tissue distribution. These isoenzyme subunit compositions are B4, B3A1, B2A2, B1A3, and A4. B4 (LDHB, LDH1, HLDH) has the highest, while A4 (LDHA, LDH5, MLDH) maintains the lowest, electrophoretic migration rate toward the anode [2,4] (Figure 1).

Except for *LDHA* and *LDHB*, the *LDHC* (LDH6, C4, is expressed in spermatocytes and in the spermatids) and the *LDHD* gene (expressed in variety of tissue types) have both also been described [5,6,7]. It is thought that *LDHA* and *LDHB* ascended from the duplication of a single LDHA-like *LDH* gene, while *LDHC* is probably a duplication of the *LDHA* gene [7].

The human LDH A-C izoenzymes have 84–89% sequence similarities, and 69–75% amino acid identities [8].

The LDHA and LDHB isoforms occupy mitochondrial compartment, plasma membrane and cytosol [9]. Although LDHA has a net charge of −6, and a higher affinity for pyruvate (it preferentially converts pyruvate to lactate and NADH to NAD^+^), whereas LDHB has a net charge of +1, and a higher affinity for lactate (preferentially converts lactate to pyruvate and NAD^+^ to NADH) [1,7], an experiment with a stable long-term knockdown of LDHA in MDA-MB-231 breast cancer cells has shown lack of changes in their glycolytic activity (defined by the production of lactic acid and ATP) [10]. According to other studies, neither LDHA nor LDHB knockout strongly reduced lactate secretion [1]. These results indicate that LDHB can spare LDHA in a majority of functions associated with the loss of LDHA [10], and both LDHA and LDHB are capable of the conversion of pyruvate to lactate [1]. Thus, a double knockdown of LDHA/B should be performed to validate in details how these enzymes (all isoforms) control pivotal events in the metabolism and production of lactic acid in tumor cells [10]. Such an experiment has been performed using double knockout (LDHA/B-DKO) in human colon adenocarcinoma LS174T cells and mouse melanoma B16-F10 cells, which resulted in fully-suppressed LDH activity and lack of lactate secretion [1].

Lactate (La^¯^), a tricarbonic anion, was discovered and initially described by Scheele [11,12,13]. It is produced in the cytosol by the reduction of pyruvate to lactate (pKa = 3.86) with the oxidation of NADH to NAD^+^, and this reaction is catalyzed by LDHA. Then, at cellular pH, lactic acid dissociates and forms a lactate anion and proton cation. Lactate (together with H^+^) can be exported from the cell (because of its anionic character, it requires a monocarboxylate transporter (MCT) to cross the cell membrane) or/and is converted to pyruvate via the LDHB-dependent reaction [11].

Overall, the knowledge of the La^−^ production has changed during decades. One might think that pyruvate is the end product of glycolysis, when the O_2_ is present, while in the case of hypoxia/anoxia, La^−^ formation is observed. However, recently a bulk of evidence points to La^¯^ production even if O_2_ is delivered to mitochondria. Thus, La^−^ is the primary end product, not only of anaerobic glycolysis, irrespective of metabolic conditions, in many cell types [13]. Moreover, in 1923 Otto Heinrich Warburg (1883–1970, Nobel Laureate, 1931) noted that tumor cells are marked by accelerated glycolysis, and consequently increased output of La^−^. According to calculations, 66–85% of glucose (even if oxygen is plentiful) is converted to La^−^, while only 5% of delivered glucose is converted to intermediates of the Krebs cycle, giving identical energy equivalent in ATP (anaerobic glycolysis vs. TCA cycle). Nowadays, this phenomenon is called the “Warburg effect”, or aerobic glycolysis [14,15,16]. Although anaerobic glycolysis generates less ATP (in terms of molar ratios between ATP and glucose) than oxidative phosphorylation (OXPHOS), this is a much faster source of ATP compared to OXPHOS, thus, the former supports cell divisions at a high rate [14].

### 1.1. LDHA and LDHB in Tumors

**Figure 2:**
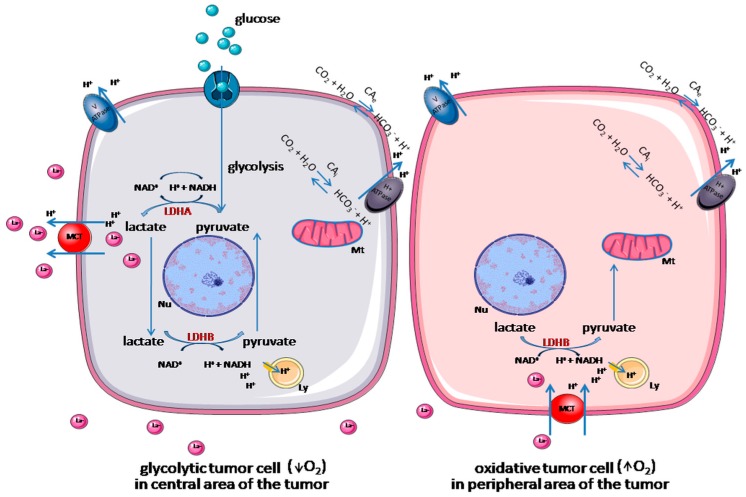
Metabolic symbiosis of tumor cells. Tumor cells, which are presented in the hypoxic area are addicted to anaerobic glycolysis. They oxidize glucose to pyruvate and lactate, which is then exported from the cell, or oxidized back into pyruvate. Then, lactate is taken up by oxidative tumor cells and oxidized to pyruvate, which fuels oxidative phosphorylation [17]. Intracellular pH homeostasis is maintained by several transporters and pumps facilitating an H^+^ efflux. Monocarboxylate transporter (MCT) facilitates the export of lactate and H^+^, while H^+^ ATPase and V ATPase extrude H^+^ from the cytosol to the extracellular matrix [20]. Ly = lysosome, Mt = mitochondrion, Nu = nucleus, LDHA = lactate dehydrogenase A, LDHB = lactate dehydrogenase B, MCT = monocarboxylate transporter, CA_i_ = intracellular carbonic anhydrase, CA_e_ = extracellular carbonic anhydrase. The main figure conception adapted from Brisson et al. (2016), and from Damaghi et al. (2013). Graphical elements adapted from Servier Medical Art.

Neoplastic cells possess a high metabolic plasticity, which allows them to choose the substrate depending on its availability. Tumor cells localized in hypoxic areas are addicted to glucose-fueled anaerobic glycolysis, whereby they oxidize glucose to pyruvate and/or lactate. In turn, oxidative tumor cells, grown in the highly vascularized areas, can use several precursor substrates, depending on their availability (for example, lactate is oxidized to pyruvate), with the aim of fueling OXPHOS [17] (Figure 2). This metabolic reprogramming with a bulk of ATP formed, contributes to multidrug resistance (MDR) [18], and is one of the reasons for increased cancer-related mortality [19].

Analysis of the LDHA expression levels in tissue sections from normal pancreas, pancreatic cystic neoplasms, as well as pancreatic intraepithelial neoplasia and pancreatic cancer, have shown LDHA is overexpressed throughout the carcinogenic process [21]. Thus, in many types of spontaneous cancers (e.g., pancreatic cancer, prostate cancer, gliomas and cutaneous melanoma metastases), an elevated LDHA expression is observed compared to normal tissues [22,23,24,25]. High levels of LDHA were confirmed also in gastric cancer (HER2 positive tumors have a significantly higher LDHA level than HER2 negative), and nasopharyngeal carcinoma [26,27]. Elevated levels of LDHA in tumor cells are considered as their metabolic adaptation to anaerobic glycolysis [23,24,25,26]. In consequence of a high rate of anaerobic glycolysis, more glucose is consumed by the tumor cells, followed by more lactic acid being formed. Therefore, a high level of lactic acid (hyperlactatamia) in the blood plasma of oncological patients, compared to healthy people (>5 mmol/L vs. <2 mmol/L) and a lower pH of their blood plasma (<7.3 vs. 7.32–7.42), are often diagnosed [12,28] and called lactic acidosis. Among three types (A, B and D) of lactic acidosis, type B is associated with highly active mitotic solid and hematological malignancies [29,30,31]. A high level of LDHA correlates with poor patient survival rates, greater tumor size, its histological grade, advanced clinical stage, Gleason scores and relapse of disease [10].

The association of LDHB with tumors is much more complex [4]. LDHB is upregulated as well as required only in certain cancer genotypes, dependent on aerobic glycolysis [32]. LDHB is silenced by promoter methylation in several cancer types, while in others it is overexpressed or amplified [4]. Analysis of the gene and protein expression of LDHB have shown its specific upregulation in basal-like/triple-negative breast cancer cell lines and tumors, as compared with luminal cancers. There is also a loss of LDHB-abolished cell proliferation in vitro, and an arrested tumor growth in vivo [32]. LDHB is upregulated in lung cancer cell lines that are characterized by RAS pathway activation and it is required for the in vivo KRAS-mutant lung tumors growth. High levels of LDHB are also observed in other lung cancer subtypes, especially in those driven by c-MET (2/2 examined cell lines) and EGFR (3/8 tested cell lines). Tumor cell lines with high levels of LDHB are more sensitive to a loss of LDHB (*p* = 0.00005) compared to LDHB low-expressing lines. It suggests that targeting LDHB may provide a broad therapeutic option for patients with lung cancer that specifically overexpress LDHB, especially given that high LDHB expression is considered as a significant predictor of shorter survival rate in patients suffering from lung adenocarcinomas [33]. Both mRNA and protein LDHB levels are elevated in polyomavirus negative Merkel cell (MCPyV)^−^ (MCC13, MCC14/2, and MCC26) carcinoma cell lines, compared to MCPyV^+^ (MKL-1, MKL-2 and WaGa) cells [34]. In contrast, in hepatocellular carcinomas, significantly low levels of LDHB compared to non-transformed tissues is observed, which predicts an unfavorable survival outcome in patients with this type of tumor [35].

As mentioned, LDHA displayed similar (high) expression levels in all tumor cells, while the LDHB level varies among different tumor cells types [36]. Interestingly, the level of LDHB can strongly differ even in the cell lines established from the same tumor type (e.g., PANC-1 vs. CaPan-1 pancreatic cells) [37]. Therefore, a significant difference in LDHA and LDHB expression levels with a predominance of LDHA expression i.e., in MDA^−^MB-231 adenocarcinoma cells, and LDHB in adenocarcinoma MCF-7 cells, can be observed in cells from the same spontaneous tumors or tumor cell lines. Such pattern of expression can contribute to divergent lactate dynamics and oxidative capacities in these tumor cells [9].

Tumor development is connected with the successive accumulation of mutations in key oncogenes, as well as tumor suppressor genes, that leads to the imbalance between cell cycle progression and cell death in favor of the first [38]. According to the information given by the Nomenclature Committee on Cell Death (NCCD), among cell death types: An intrinsic apoptosis, extrinsic apoptosis, mitotic catastrophe, mitochondrial permeability transition (MPT)-driven necrosis, necroptosis, ferroptosis, pyroptosis, parthanatos, entotic cell death, NETotic cell death, autophagy-dependent cell death, lysosome-dependent cell death, cellular senescence and immunogenic cell death, are mentioned [39]. However, also during tumorigenesis, metabolic and therapeutic stresses can cause a series of adaptive responses and suicide signals in tumor cells.

The sum of these programmed adaptations and death signals determines the fate of the cell: cell death or cell survival [40]. On the long list of factors that regulate tumor cell death, LDHA and LDHB are mentioned.

### 1.2. LDHA and LDHB Regulation

A variety of physiological signals regulate LDH izoenzymes at transcriptional, post-transcriptional and post-translational levels [41]. Transcriptionally, LDHA is regulated by forkhead box protein M1 (FOXM1), which binds directly to four putative FOXM1-binding elements in the *LDHA* gene promoter region. Thus, an increased expression of FOXM1 upregulates the expression of LDHA at both mRNA and protein level. FOXM1-LDHA signaling functions as a stimulator of glycolysis, and promotes cancer progression through promotion of its growth and metastasis [42,43]. Also c-Myc, an oncogenic transcription factor, is able to directly transactivate the LDHA promoter, and increases LDHA expression. Elevated LDHA levels associated with overexpression of c-Myc are necessary for neoplastic transformation [44]. Transcriptional induction of LDHA can also be caused by hypoxia, which is often a consequence of lower oxygen delivery vs. consumption mismatch, occurring when tumor cell proliferation outstrips neoangiogenesis during tumor growth [45,46]. Both hypoxia-inducible factors (HIF-1α and HIF-2α) interact with functional hypoxia-responsive element D (HRE D; 5 -G/ACGTG-3) in the LDHA promoter, and bind to LDHA at 89 bp under the hypoxic condition [45]. Indirectly, TGF-β, possibly via stabilization of HIF-1α, upregulates LDHA [47]. Also Jumonji C domain 2A (JMJD2A), a histone demethylase, combines with the LDHA promoter region and positively regulates LDHA expression at the level of transcription [27]. *LDHA* expression can be also induced by estrogen, which acts on the *LDHA* promoter [48]. LDHA mRNA at the transcriptional and post-transcriptional levels can be also modulated by cyclic adenosine monophosphate (cAMP) and the protein kinase A [41]. An increased LDHA expression in multiple myeloma cells can be regulated by peroxisome proliferator-activated receptor gamma (PPAR-γ) coactivator 1-beta (PGC1β). This regulation is through a PGC1β-mediated increase of RXRβ binding capacity to the LDHA promoter [49].

In the post-transcriptional regulation, a miR-200b inversely correlates with the LDHA level in gliomas. Thus, repression of LDHA by miR-200b suppresses the glycolysis, cell proliferation, as well as invasion of glioma cells [22]. Similar results emphasize the role of miR-34a, miR-34c, miR-369-3p, miR-374a and miR-4524a/b on the LDHA repression in colorectal cancer [50] or breast cancer cells (miR-34a) [51]. Also miR-199a-3p can inhibit LDHA expression (by downregulating the Specificity protein 1 (Sp1)-transcription factor), which supports the critical contribution of a miR-199a-3p/Sp1/LDHA axis to aerobic glycolysis in cancer cells [52].

LDHA regulations through post-translational modifications include acetylation. LDHA is acetylated at lysine 5 (K5) in pancreatic cancer cells, which reduces LDHA catalytic activity and decreases LDHA protein level. The K5-acetylated LDHA is recognized by the HSC70 chaperone, and delivered to lysosomes for its degradation. Because replacement of endogenous LDHA with an acetylation mimetic mutant leads to a decrease of cancer cell proliferation and migration, LDHA acetylation plays a critical role in cell growth. Moreover, K5 acetylation of LDHA is reduced and accompanied by increased LDHA protein levels in early and late stages of pancreatic cancers, which suggests a possible role of K5 acetylation in pancreatic cancer initiation, but not progression [53]. Interestingly, Tyr10-phosphorylation by upstream kinases, HER2 and Src is needed for LDHA activation, and provides pro-invasive, antianoikis and prometastatic values to cancer cells. Phosphorylation at Tyr10 activates LDHA and may generate NAD^+^ to sustain aerobic glycolysis and directly correlates with activities of several oncogenic tyrosine kinases (e.g., BCR/ABL, FGFR1, FLT3-ITD and JAK2) [54]. Tyr10 constitutive phosphorylation decreases tumor cells proliferation, as well as ATP levels under hypoxic conditions. It also diminishes tumor growth in xenograft nude mice [55]. At the protein level, LDHA can be regulated by fibroblast growth factor receptor 1 (FGFR1). FGFR1 directly phosphorylates the four tyrosine residues of LDHA, which promotes the stability of LDHA and increases its enzymatic activity in pancreatic cancer cells [56].

Compared to LDHA, the knowledge related to the LDHB regulation is not well established. Among positive regulators of LDHB expression the mammalian target of rapamycin complex 1 (mTORC1) is mentioned. LDHB transcription is also directly stimulated by the signal transducer and activator of transcription 3 (STAT3), a key tumorigenic driver in many cancers. Thus, knocking down STAT3 leads to the LDHB reduction [57]. Also High-mobility group box 2 (HMGB2) is involved in the transcriptional regulation and activation of LDHB [58]. On the contrary, FGFR1 suppresses LDHB transcription through the promoting DNA methylation in the LDHB promoter via inducing an expression of Tet1, a DNA-binding protein [56]. However, this promoter methylation of LDHB is an epigenetic abnormality, not a genomic alteration [59]. LDHB mRNA and protein levels are repressed followed by overexpression of miR-375 [60] and increase after suppression of miR-375, which supports the notion that LDHB is a target of tumor cells miR-375 [34].

## 2. LDHA, LDHB and Lactic Acid in the Cell Death of Tumor Cells

### 2.1. Apoptosis

Apoptosis, a physiological cell suicide program, maintains cell number and cellular positioning within tissues comprised of different cell compartments. It is essential for the regulation of development, keeping of homeostasis and the prevention of tumorigenesis [61,62]. However, evading apoptosis or resisting cell death is thought of as a hallmark of cancer. It represents an important mechanism in tumor resistance to oncological therapies [62,63].

Cytokines that trigger apoptosis include all types of interferons (naturally-secreted glycoproteins) [64]. There is a higher proportion of anti-tumor effector cells (CD8^+^ T and NK cells) in Ldha^low^ tumors, compared to control tumors grown in C57BL/6 mice. As a consequence of elevated numbers of these cells in tumor, an increased amount of interferon gamma (IFN-γ) and granzyme B is observed. It indicates, that lactic acid prevents tumor infiltration by IFN-γ and granzyme B producing T and NK cells, which promote tumor immune evasion and growth [25].

Exposure to cellular stressors can trigger p53 tumor suppressor (a sequence-specific transcription factor), which induces cell growth arrest or apoptosis [65]. Cells committed to die via p53-dependent apoptosis typically follow the mitochondrial pathway, however p53 can also modulate cell death through death receptors. The key contribution of p53 to apoptosis is the ability to activate the transcription of various proapoptotic genes, including those encoding members of the *BCL-2* family, such as the BH-3 only proteins *BAX*, *NOXA*, and *PUMA* [66]. LDHA depletion induces apoptosis in p53 wild-type, mutant and p53-null cancer cells, thus irrespective of p53 status. However, targeting LDHA provides a p53-dependent mechanism, by which the NADH:NAD^+^ is balanced in cancer, but not in non-transformed cells. It causes a p53-dependent increase of cancer cell sensitivity to the redox-dependent anticancer drugs. Elevated cancer cell ratio of NADH/NAD^+^ can impact enzymes utilizing NAD^+^ and NADH, and has lethal consequences [67]. NAD^+^ depletion mediates PARP-1-induced cell death. Also mitochondrial permeability transition and apoptosis-inducing factor (AIF) translocation links NAD^+^ depletion to cell death. Significant increase in PARP-1 activity, followed by AIF-mediated cell death, are observed as a consequence of sirtuins (SIRTs; NAD^(+)^-dependent enzymes) deficiency [68].

Because LDHA silencing can alter cancer cell metabolism from glycolysis to mitochondrial respiration (through preferring the entry of pyruvate into mitochondria), lack of LDHA enhances oxygen consumption (in both p53^+/+^ and p53 ^-/-^ cancer cells), which results in elevated level of mitochondrial reactive oxygen species (ROS) (~2.3 fold; *p* < 0.001 in LDHA deficient HCCLM3 hepatocellular carcinoma cells compared to control cells) [67,69,70,71]. As the ROS are powerful regulators of Ca^2+^ signaling, knocking down the LDHA causes an increase of intracellular levels of Ca^2+^ (~2.9-fold; *p* < 0.001), which may be involved in triggering apoptosis, inter alia, via an activation of apoptotic endonucleases [72,73]. LDHA depletion causes changes, not only in mitochondrial functions and metabolism, but also in their morphology. Mitochondria are abnormally swollen, their matrices are pale and cristae disorganized. As a consequence, the mitochondrial membrane potential is decreased [70].

**Figure 3:**
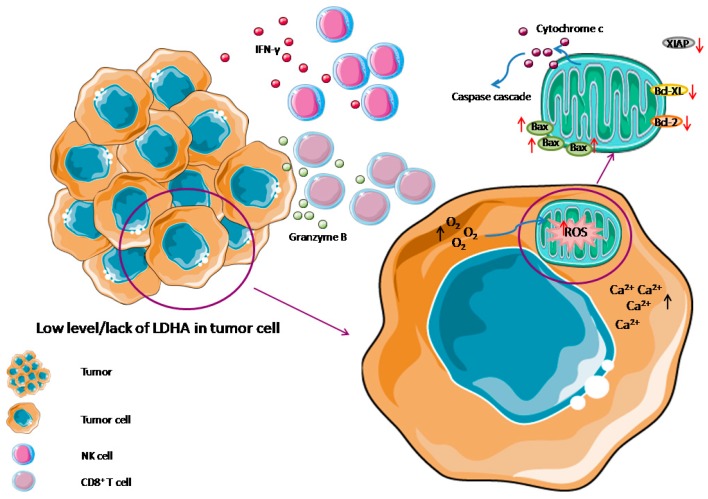
The role of LDHA in the apoptosis of a tumor cell. IFN-γ = interferon gamma, ROS = reactive oxygen species. Graphical elements adapted from Servier Medical Art.

All of these changes indicate that LDHA silencing or using an LDHA inhibitor induces an apoptosis ratio in cancer cells via the mitochondrial pathway, measured by the percentage of apoptotic cells (~3.3-fold increase; *p* < 0.001) or by estimating the sub-G1 cell fraction [72,74]. Knocking down LDHA enhances the cytochrome *c* release (the amount released is 5.38 ± 0.71 ng·mL^−1^ at 48 h LDHA shRNA Lenti treatment of hepatocellular carcinoma cells) from the mitochondria to the cytoplasm, providing a key signal initiating the irreversible death sequence [70,72,75]. This sequence includes an elevated level of cleaved caspase 9—an apoptosis initiator, which then cleaves an executioner procaspase-3 and procaspase-7, followed by PARP cleavage (PARP fragments are described as an indicator of apoptosis) [70,76]. Higher levels of both cleaved procaspase-3, procaspase-7 and cleaved PARP, are also observed in LDHA-defective cancer cells [24,70,72]. Moreover, LDHA inhibition results in an increase of the Bax level, which insertion into the mitochondrial membrane induces the release of cytochrome *c* and the induction of apoptotic cell death [74,77]. At the same time, lack of LDHA results in decreased expression of the proteins involved in apoptosis inhibition: Bcl-2, Bcl-XL, which both inhibit mitochondrial cytochrome *c* release, as well as XIAP [23,70,74,78]. It acts to suppress accidental cell death via potent inhibition of procaspase cleavage [79]. The possible mechanism of LDHA action in the apoptosis of a tumor cell is illustrated in Figure 3.

The significance of the LDHB role in tumor cell death, including apoptosis, is not well understood. LDHB inhibition in MCPyV-MCC cells causes increased cleavage of PARP, which leads to apoptosis [34]. Knockdown of LDHB enhances taxol-induced apoptosis, confirmed by cytochrome *c* release, activation of caspase-3 and -7, and reduced expression of Bcl-2. This pro-apoptotic effect can be reversed by LDHB implementation [36]. Some data, as knocking down *LDHB*, showed no apparent effect (similarly to LDHA) on apoptosis induction in cancer cells and silencing of the *LDHB* gene does not increase the cellular NADH/NAD^+^ ratio in these cells [36,67].

### 2.2. Autophagy

The term “autophagy” derives from the Greek meaning “eating of self”. It is a self-degradative process important for balancing sources of energy at critical times, in development and in response to nutrient deprivation [80]. It maintains not only cellular homeostasis, but also viability under hostile conditions [81]. 

Autophagy plays also an important role in the clearing of damaged or superfluous organelles (such as mitochondria), removing misfolded or aggregated proteins and eliminating intracellular pathogens [80]. In addition, heterophagy acts in various aspects of immunity, for example in the elimination of invasive microbes and its participation in antigen presentation [81]. Thus, autophagy is generally thought of as a survival mechanism, however its deregulation has been linked to non-apoptotic cell death [80]. Moreover, autophagy initially prevents or at least delays tumor formation (by protecting the cell from potentially damaging species that might lead to mutational and carcinogenic damage), but once tumor formation has progressed, autophagy can protect the tumor cells from environmental injury, and it also supports tumorigenesis [82,83]. Thus, autophagy is considered as a double-edged sword, suppressing cancer initiation, or promoting the growth of established cancers [84]. Resistance to chemotherapeutic agents is a major problem in oncological treatment, which limits the effectiveness of anticancer drugs, and increases cancer-related mortality. A variety of factors (host factors, specific genetic or epigenetic alterations in the cancer cells), contribute to drug resistance [19]. Interestingly, autophagy is also involved in MDR development, as well as radioresistance [85,86]. When cancer cells are subjected to stressful conditions (imposed upon chemotherapy and/or radiotherapy), autophagy is rapidly upregulated. It maintains metabolic homeostasis, and ensures that cell growth is appropriate to its changing microenvironmental conditions, through reduced growth and increased catabolic lysis of unnecessary or excessive proteins and/or organelles [19]. The induction of autophagy is frequently thought to perform an additional cytoprotective function by preventing cell death through apoptosis [87]. Nutrient deprivation, which widely exists in solid tumors because of the poor blood supply and fast cell division rate, is a crucial activator of autophagy. Low-nutrient conditions drive cancer cells to utilize glycolysis to produce ATP, which increases the Warburg effect [88]. Knockdown of the ATG7 (protein important in ubiquitin-like conjugation system for the autophagosome elongation) not only decreases glucose uptake and lactate secretion by tumor cells (suggesting a reduction in anaerobic glycolysis), but also sensitizes chronic myeloid leukemia progenitor cells to tyrosine kinase inhibitor-induced cell death [89].

However, until today the role of LDHA and LDHB in autophagy is unclear. Little is known as to whether autophagy can be induced by LDHA or LDHB inhibition, and what consequences (survival or death) brings about the cell to activation of this process [90].

The experiments with targeting LDHA and LDHB showed that LDHB, but not LDHA, controls lysosomal activity and basal autophagic flux of cancer cells. Inhibition of LDHA activity using targeting siRNA, did not alter the autophagic flux determined with LC3-II and optineurin in SiHa human cervix adenocarcinoma cells, as well as did not decrease their number. But silencing LDHB-induced leupeptin-sensitive LC3-II protein accumulation in SiHa cells, represents a potent inhibition of the autophagic flux, and is associated with an accumulation of optineurin, an autophagic substrate. Moreover, silencing LDHB had an antiproliferative effect on cancer cells. Similar results were obtained using HeLa adenocarcinoma cells, in which siLDHB caused a decrease of LC3-II flux and prevented optineurin degradation. Taken together, these data demonstrate that LDHB, but not LDHA, controls the basal autophagic flux of oxidative cancer cells, and silencing LDHB inhibits basal autophagy, cancer cell proliferation, and also leads to apoptosis. Worth mentioning, siLDHB did not repress the growth and autophagic flux in non-malignant BJ, HUVEC, MCF10A cells. Although siLDHB has no additive effect on the genetic disruption of autophagy by siULK1 (which targets an early step of autophagy), and on autophagy inhibition by chloroquine (which inhibits lysosomal activity), the controlling of autophagy by LDHB involves its participation in autophagic vacuole maturation. Moreover, H^+^ generated during the reaction catalyzing by LDHB (lactate and NAD^+^ are converted to pyruvate and NADH and H^+^, respectively) promotes V-ATPase-dependent lysosomal acidification [17]. The post-translational mechanism, by which LDHB is regulated during autophagy in cancer cells, implicates sirtuin 5 (SIRT5). SIRT5 is a binding partner for LDHB. It deacetylates LDHB at lysine-329 (a major acetylation site of LDHB, K329), thereby promoting its enzymatic activity [91].

Deacetylated LDHB increases the autophagy of tumor cells, helps lysosomal acidification and autolysosomal maturation, while silencing or the inhibition of SIRT5 leads to LDHB acetylation at K329, which inhibits its proautophagic activity [91].

**Figure 4:**
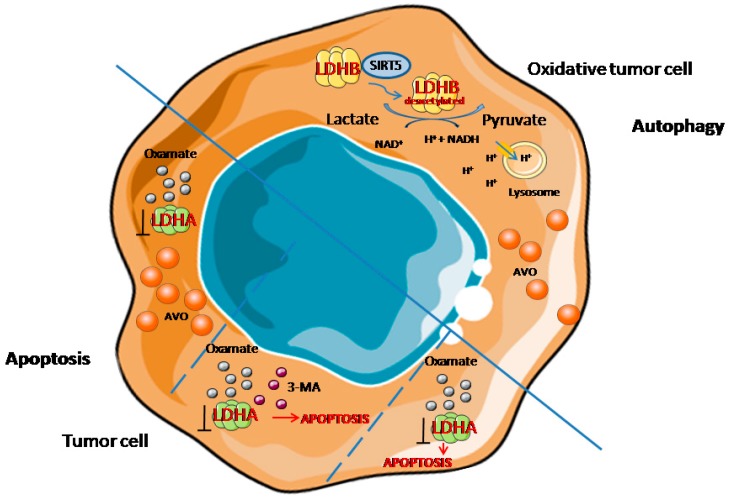
The role of LDHA and LDHB in autophagy/apoptosis of tumor cells. 3-MA = 3-Methyladenine, AVO = acidic vacuolar organelles, LDHA = lactate dehydrogenase A, LDHB = lactate dehydrogenase B, SIRT5 = Sirtuin 5. Graphical elements adapted from Servier Medical Art.

Oxamate is an inhibitor of LDHA, which acts via competition with pyruvate for its binding site of the enzyme. It has been widely used in many studies as a promising anticancer drug that interrupts aerobic glycolysis. Furthermore, 24 h oxamate treatment of gastric cancer cells caused an increase of LC3-II, as well as P62 measured by Western blot techniques. Moreover, evaluation of recruitment of LC-II to autophagosomes in response to oxamate treatment with using a pEGFP-LC3 plasmid showed a punctate LC3 pattern in oxamate-treated cells in cytoplasm, compared to diffuse and weak LC3 punctae dots in untreated control cells. Ultrastructure analysis of cancer cells treated by oxamate clearly demonstrated the presence of numerous membrane-associated vacuoles located in the cytoplasm. These changes indicate that oxamate induces autophagy in gastric cancer cells. Moreover, inhibition of oxamate-induced autophagy caused an increase in the number of apoptotic cells, which points to autophagy as a cytoprotective mechanism. The induction of autophagy is connected with the increased ROS generation in gastric cancer cells following 24h treatment with oxamate. Thus, oxamate induces autophagy, while its molecular mechanism implicates a repressed PI3K-Akt-mTOR-p70s6k signaling pathway. Treating cancer cells with oxamate leads to phospho-Akt inhibition, followed by the down-regulation of downstream phospho-mTOR and phospho-p70s6k [84]. In agreement with the results given by Zhao et al. (2015), 24h of oxamate treatment led to the acidic vacuolar organelles (AVO) formation, and changes in LC3 lipidation also in non-small cell lung cancer A549 cells. Surprisingly, different effects of LDHA inhibition by oxamate were described in other similar H1395 cell lines, in which oxamate caused an increased number of apoptotic cells. In the case of A549 cells, apoptosis was initiated only by the addition of autophagy inhibitor 3-MA with oxamate. Thus, in this type of tumor cells, a different response to the LDHA inhibition is observed, which provides a novel insight into the signaling pathway, shifting cancer cells towards either apoptosis or autophagy [90]. The possible mechanisms of LDHA and LDHB action in autophagy/apoptosis of tumor cells is illustrated in Figure 4.

The role of LDHA and LDHB in tumor biology is more complex than was initially expected. This enzyme ensures not only metabolic plasticity of neoplastic cells, which allows them to adapt to the hostile environmental or cellular conditions, but it also regulates cell death. Thus, targeting LDHA or/and LDHB can create the new opportunity to combat cancer cells.

## Abbreviations

3-MA	3-Methyladenine
AIF	Apoptosis-inducing factor
AKT	AKT Serine/Threonine Kinase
AMP	Adenosine monophosphate
ATP	Adenosine triphosphate
ATPase	Adenosine triphosphatase
AVO	Acidic Vacuolar Organelles
BAX	BCL2 associated X protein
BCL-2	B-cell lymphoma 2
BH-3	Interacting-domain death agonist
Ca^2+^	Calcium ion
EGFR	Epidermal Growth Factor Receptor
FGFR1	Fibroblast Growth Factor Receptor 1
FOXM1	Forkhead Box Protein M1
H^+^	Hydrogen ion
HER2	Human Epidermal Growth Factor Receptor 2
HIF	Hypoxia-inducible factor
HMGB2	High-mobility group box 2
IFN-γ	Interferon gamma
JMJD2A	Jumonji C Domain 2A
KRAS	KRAS proto-oncogene
La^−^	Lactate
LC3-II	Microtubule-associated protein 1A/1B-light chain 3 (LC3) conjugated to phosphatidylethanolamine
LDH	Lactate dehydrogenase
LDHA	Lactate dehydrogenase A
LDHB	Lactate dehydrogenase B
LDHC	Lactate dehydrogenase C
LDHD	Lactate dehydrogenase D
MCC	Merkel cell carcinoma
MCPyV	Merkel cell polyomavirus
MCT	Monocarboxylate Transporter
MDR	Multidrug resistance
miR	microRNA
MPT	Mitochondrial permeability transition
mTOR	Mammalian Target of Rapamycin
NAD^+^	Nicotinamide adenine dinucleotide (oxidized form)
NADH	Nicotinamide adenine dinucleotide (reduced form)
NCCD	Nomenclature Committee on Cell Death
NOXA	Phorbol-12-myristate-13-acetate-induced protein 1
OXPHOS	Oxidative phosphorylation
PGC1β	Peroxisome proliferator-activated receptor-gamma coactivator 1 – beta
PI3K	Phosphoinositide-3-kinase
PPAR-γ	Peroxisome proliferator-activated receptor-gamma
PUMA	p53 upregulated modulator of apoptosis
ROS	Reactive oxygen species
SIRT	Sirtuin
SIRT5	Sirtuin 5
Sp1	Specificity protein 1
STAT3	Signal Transducer and Activator of Transcription 3
